# Measuring Optimal Reading Experiences: The Reading Flow Short Scale

**DOI:** 10.3389/fpsyg.2018.02542

**Published:** 2018-12-13

**Authors:** Birte A. K. Thissen, Winfried Menninghaus, Wolff Schlotz

**Affiliations:** ^1^Department of Language and Literature, Max Planck Institute for Empirical Aesthetics, Frankfurt am Main, Germany; ^2^Labs and Methods, Max Planck Institute for Empirical Aesthetics, Frankfurt am Main, Germany; ^3^Institute of Psychology, Goethe University Frankfurt, Frankfurt am Main, Germany

**Keywords:** flow, fiction reading, Reading Flow Short Scale, validity, reading pleasure

## Abstract

In transferring the concept of flow to the context of fiction reading a new approach to understanding the evolvement of reading pleasure is provided. This study presents the Reading Flow Short Scale (RFSS), the first reading-specific flow measurement tool. The RFSS was applied to 229 readers via online survey after 20 min of reading in self-selected novels. In a systematic analysis of psychometric properties, the RFSS’ factorial structure, reliability, and associations with theoretically related constructs were examined. As expected, the RFSS showed a two-factor structure, positive correlations with variables related to reading pleasure and flow, and an inverted U-shaped association with perceived fit between reader skills and text challenge. Comparisons of confirmatory factor analysis model confirmed that RFSS items loaded on different latent variables than items assessing other narrative engagement concepts, namely presence, identification, suspense, and cognitive mastery, and hence distinctly capture flow states in fiction reading. In sum, our findings indicate that the RFSS is a useful instrument for assessing flow states in fiction reading, thereby enriching the portfolio of measurement instruments in reading research.

## Introduction

Considering the growing body of empirical evidence on positive effects of fiction reading (Mar et al., 2011; Kidd and Castano, 2013; Vezzali et al., 2015), there is still relatively little consensus regarding the mental mechanisms involved in making reading itself an inherently rewarding experience. Reading pleasure has been discussed to be mediated by the reader’s change of consciousness (Nell, 1988), which can occur both in response to text-inherent incitements and the activity of reading itself. Thus, on the one hand, reading fictional texts can elicit specific pleasure-related states in the reader as a direct reaction to engagement with certain story elements. Amongst the most prominent concepts of pleasure-related narrative engagement are *presence* (Lee, 2004), *suspense* (Zillmann, 1996), *identification* (Cohen, 2001), and *cognitive mastery* (Oliver and Raney, 2011). While presence states are defined as the sensation of being in the story world, states of heightened reader suspense pertain to the anticipation of emotionally charged story events. A state of identification is characterized by the internalization of story-characters’ feelings and thoughts, and cognitive mastery states arise from the sense of retrieving meaning, truth and purpose from the story. Depending on the narrative the reader engages with, varying degrees and combinations of presence, suspense, identification, and cognitive mastery might occur and contribute to making the reading experience pleasurable.

On the other hand, the activity of reading itself can cause the reader to enter a pleasurable state of heightened absorption or even *flow* (Csikszentmihalyi, 1975). Flow states are defined as the optimal experience of being fully engaged in an activity and have been used throughout the field of positive psychology as a theoretical framework for intrinsic enjoyment. Whenever the degree of challenge in a given activity perfectly matches a person’s individual skill level, the person will experience flow (Csikszentmihalyi, 1975). Optimally balanced skills and challenges and flow experiences have been shown to increase subjective involvement and enjoyment of activities (i.e., Keller and Bless, 2008; Keller and Blomann, 2008), supporting the conceptualization of flow as a motivator for repeated activity engagement (Csikszentmihalyi, 1975). Furthermore, flow has been positively associated with personal preferences and self-efficacy in regard to a certain activity (Rheinberg et al., 2003).

Several authors have offered theoretical considerations on flow and enjoyment of narratives (i.e., Muth, 1996; Busselle and Bilandzic, 2008; Weber et al., 2009), discussing flow during reading as a key element of reading pleasure. While engagement with a specific narrative can lead to different pleasure-related states, such as presence, identification, suspense, and cognitive mastery, engagement with the activity of reading itself, or more precisely with the activity of constructing a mental model of the story (Busselle and Bilandzic, 2008), can lead to a flow state. Reading pleasure should therefore be considered a multi-dimensional experience, with flow as one of its components and a potential mediator for others. In contrast to other concepts discussed as being part of a pleasurable reading experience, including the formerly mentioned ones, the flow concept comes with the advantage of an underlying comprehensive theoretical model from which precise predictions can be derived. Thus, predicting flow experiences in readers based on their perceived balance of text challenge and reader skills can ultimately help to make individual reading pleasure more predictable.

In order to empirically research the role of flow states in fiction reading, a psychometrically tested measurement device is needed, which assesses flow states in this specific context. To the best of our knowledge, only two studies have investigated flow experiences in fiction reading empirically so far. Massimini et al. (1988) conducted a meta-analysis of five studies surveying flow experiences in everyday life by means of a general flow measure, finding that reading ranked as the most frequently self-reported flow activity. Applying the same general flow measure to a sample of regular fiction readers instructed to fill it out with the activity of reading in mind, McQuillan and Conde (1996) reported flow to be most likely for reading fictional texts, texts related to personal interests, and for reading during leisure time. However, the flow measure employed in both studies, the Flow Questionnaire (FQ; Csikszentmihalyi and Csikszentmihalyi, 1988), has been criticized in recent flow research in terms of its conceptual and psychometric properties, with major points of critique being its lacking differentiation between high or low levels of flow, its systematic underestimation of flow prevalence in specific contexts, and its proneness to distortion through memory effects (Moneta, 2012). To overcome such limitations, flow research has widely adopted the so called componential measurement approach (Moneta, 2012), which is based on Csikszentmihalyi’s (1975) conceptualization of flow as a multi-componential state of mind. Thus, flow states are characterized by nine components (Csikszentmihalyi, 1975; Jackson and Marsh, 1996): (1) *merging of action and awareness*, (2) *attentional focus*, (3) *loss of self-awareness*, (4) *sense of control or competence*, (5) *perception of coherent, non-contradictory demands*, (6) *intrinsic enjoyment*, (7) *distorted sense of time*, (8) *perception of clear goals*, and (9) *perception of unambiguous feedback.* Componential flow measures collect self-report ratings on how far individuals experience each of these flow components directly after engagement in an activity, from which subsequently a joint flow score is calculated (Jackson and Eklund, 2002; Rheinberg et al., 2003). Flow scales of this type have been shown to significantly outperform other measurement approaches, including the FQ, in terms of psychometric properties (Moneta, 2012).

In the last decades, several componential flow scales have been developed, both for flow measurement in specific contexts, such as work (Bakker, 2008) or internet surfing (Novak and Hoffman, 1997), and for flow measurement across activities (Jackson and Marsh, 1996; Rheinberg et al., 2003; Jackson et al., 2008). One particularly prominent general flow scale in European flow research is the Flow Short Scale (FSS; Rheinberg et al., 2003), a brief 13-item measure, with three supplemental items assessing perceived balance of skills and challenges. The FSS has been shown to have good psychometric properties (α = 0.90), a stable 3-factor structure comprising Absorption, Smooth Processing, and Concern, and the expected associations with theoretically related constructs such as skills-challenge balance, performance, and self-efficacy, supporting the scale’s validity (Rheinberg et al., 2003). The original German-language scale has been translated to different languages (for an overview see Rheinberg, 2015), including English (Engeser and Rheinberg, 2008), and applied across a wide range of flow activities, such as marathon running (Schüler and Brunner, 2009), computer gaming (Weibel and Wissmath, 2011), and learning (Vollmeyer and Rheinberg, 2006).

Even though the FSS has been designed as a general flow scale, its applicability to fiction reading is limited as the wording of certain items implies engagement in a motoric, performance-related, and competitive activity, which makes sense for most typically studied flow contexts, but not for this one. Particularly, the subdimension of Concern, which measures an absence of fear of failure, does not match the non-performance-related context of fiction reading. Thus, fiction reading can to some degree be seen as a specific flow context, in which optimal challenge levels primarily refer to stimulation instead of achievement and in which the activity itself is primarily mental instead of motoric. While these characteristics do not directly interfere with the applicability of the various flow components themselves to the context of fiction reading, they do interfere with certain items intended to measure them on general scales such as the FSS. Therefore, any valid measure of flow in fiction reading has to be specific in the sense that it needs to be adapted to the special characteristics of this activity.

We know of only one scientific endeavor to formulate reading-specific flow items, which were constructed as part of a bigger reading-experience measure (Appel et al., 2002). However, these items substantially deviate from the componential measurement approach as they only measure intrinsic enjoyment and perception of coherent demands, neglecting other flow components. Moreover, since flow was not a focus of the corresponding study, the items were not systematically tested for validity. Given the lack of conceptually and psychometrically sound reading-specific flow measures, we developed a new instrument, the Reading Flow Short Scale (RFSS), by adapting the FSS to fiction reading. In the current study, (1) we investigated the RFSS’ factorial structure and reliability, and (2) further explored its construct validity in terms of associations with theoretically flow-related constructs and its differentiability from other pleasure-related concepts of narrative engagement.

## Materials and Methods

### Scale Development

We derived the RFSS from the FSS by dropping its 3-item subdimension Concern, which applies to competitive flow activities only, and by rephrasing the 10 remaining items on the subscales Absorption and Smooth Processing to ensure good fit to the context of fiction reading. For items on the Absorption subdimension this was sufficiently achieved by integrating specific references to reading into the item wording (i.e., “I did not notice time passing.”/“I did not notice time passing during reading.”). The items on the Smooth Processing subdimension partly required more throughout rewording (i.e., “The right thoughts and movements occured on their own account.”/”Thoughts, emotions, and images emerged automatically and spontaneously, inspired by what I was reading.”). All rephrased items were submitted to flow experts for approval. Table 1 shows the final 10 RFSS items, to be answered on a seven-point Likert-type scale ranging from *strongly disagree* to *strongly agree*.

**Table 1:** RFSS items with mean scores, factor loadings and communalities for exploratory factor analysis with Geomin rotation.

	Item	*M (SD)*	Factor loadings		*h*^2^
				
			Factor 1	Factor 2	
1	I felt optimally challenged during reading.	5.39 (1.46)	**0**.**48**	-0.01	0.23
2	I read this text smoothly and fluently.	5.76 (1.35)	0.11	**0**.**60**	0.45
3	I did not notice time passing during reading.	5.34 (1.58)	**0**.**59**	0.12	0.43
4	I had no problem to concentrate during reading.	5.50 (1.50)	0.35	0.34	0.35
5	My mind was totally clear during reading.	5.43 (1.47)	0.33	0.34	0.35
6	I was completely immersed in what I was reading.	5.36 (1.36)	**0**.**82**	0.03	0.71
7	Thoughts, emotions, and images emerged automatically and spontaneously, inspired by what I was reading.	5.50 (1.39)	**0**.**46**	0.07	0.25
8	I knew on every page that I was able to grasp the story.	6.16 (1.22)	-0.04	**0**.**82**	0.64
9	I had the feeling that I understood everything during reading.	4.58 (1.62)	**0**.**79**	-0.01	0.54
10	During reading I became so oblivious that I became completely unaware of myself.	6.07 (1.19)	0.02	**0**.**73**	0.56
% of variance			25.00	20.00	


*Factor loadings >0.40 are in boldface. Percentage variance is post-rotation. Items 4 and 5 were later removed from the RFSS due to cross-loadings. All items were adapted from the Flow Short Scale by F. Rheinberg et al. (2003).*

### Design and Procedure

An online survey was set up using Unipark/EFS Survey and made accessible from April to July, 2016. In the survey, participants were instructed to read on in a self-selected novel for 20 min. When reading time was over, a timer embedded in the online survey rang a signal. Participants then completed the RFSS, items assessing perceived skills-challenge balance, convergent measures assessing reading and reader variables and previous reading-related flow experiences, as well as discriminant measures assessing presence, identification, suspense, and cognitive mastery.

After survey completion, respondents could enter a lottery to win one of 70 online book vouchers worth 20€ each. The survey itself was anonymous; participation was voluntary and could be withdrawn at any time. All procedures were ethically approved by the Ethics Council of the Max Planck Society and were undertaken with informed consent of each participant.

### Participants

Participants were recruited by disseminating flyers in local bookstores, during public readings, and in undergraduate literature courses at the local Goethe University, as well as by sharing the survey link on Facebook and in reading forums. Mean duration for survey completion was 45.3 min, including 20 min during which participants read on in a novel they were currently reading. The most frequent novel genres read in the sample were *Fantasy* (14%), *Crime/Thriller* (14%), *Social Novel* (13%), *Psychological Novel* (12%), and *(Melo-)Drama* (8%). To ensure its potential for reader engagement, the novel read in the study had to meet the criteria of being self-selected, already finished halfway, written in a language the participant is fluent in, and telling an unfamiliar story. For not meeting these criteria, 98 readers were excluded before they could start the survey. Another five participants were excluded afterward because their answers to control questions indicated non-attentive reading or careless response behavior (Meade and Craig, 2012).

The final sample comprised 229 participants, most of them being female (*n* = 181; 79%). The sample covered an age range between 18 and 81 years (*M* = 35.6, *SD* = 15.0) and showed a relatively high educational background: Thus, more than half of the participants (*n* = 122; 53%) held a graduate degree, another 5% (*n* = 12) a postgraduate degree, and 85 persons (37%) a general qualification for university entrance.

### Measures

#### Convergent Measures

##### Reading and reader variables

Reading pleasure as well as motivation to read on and to read another similar story were assessed using single items on five-point Likert-type scales. Participants answered additional single items measuring general affinity for fiction reading and reading frequency in regard to fictional texts. Beliefs concerning one’s own ability to comprehend and enjoy fictional texts were measured by means of a 4-item reading-specific self-efficacy scale (see Table 2; McDonald’s ω = 0.62 [0.49, 0.70]), which had been developed on the basis of two general self-efficacy scales (Engeser, 2005; Beierlein et al., 2012). All ratings and rating scores were expected to be positively associated with flow and hence positively correlated with the RFSS flow score due to the close conceptual link between flow and intrinsic enjoyment, repeated activity engagement, personal preference, and self-efficacy.

**Table 2:** Items used for assessing presence, identification, suspense, cognitive mastery, and reading self-efficacy.

Construct	Item
Presence	When I stopped reading, I felt like I came back to the “real world” after a journey.
	During reading, my mind was in the room, not in the world created by the novel (reversed).
	During reading, my body was in the room, but my mind was inside the world created by the story.
	The story created a new world, and then that world suddenly disappeared when I stopped reading.
	At times during reading, the story world was closer to me than the real world.
Identification	I was able to understand the events in the story in a manner similar to that in which the protagonists understood them.
	I think I have a good understanding of the story’s protagonists.
	I tend to understand the reasons why the protagonists do what they do.
	While reading the story, I could feel the emotions the protagonists portrayed.
	During reading, I felt I could really get inside the protagonists’ heads.
	At key moments in the story, I felt I knew exactly what the protagonists were going through.
	During reading, I wanted the protagonists to succeed in achieving their goals.
	When the protagonists succeeded I felt joy, but when they failed, I was sad.
Suspense	During reading, I was really thrilled to see how the story would go on.
	I could not wait to start the next page to find out what would happen next in the story.
	I found the story so gripping, that I was hesitant to stop reading.
	It was exciting for me to imagine how the story would go on during reading.
	During reading I developed hopes and fears about how the story might end, and I was curious to find out whether they were true.
Cognitive mastery	While reading this story, I sensed something that I could not find a way to express.
	After reading this story, I felt that my understanding of life had been deepened.
	To me, the story seemed to have a deeper meaning, which I tried to figure out during reading.
	I found that reading the story was thought-provoking for me.
	During reading, I felt that I was learning new things that would enrich my view of the world.
Reading self-efficacy	If a book is interesting to me, I don’t care how hard it is to read.
	If a book is interesting to me, I will read it even if it is long.
	If I don’t immediately find an approach to a story, I can rely on my abilities to comprehend and feel stories.
	Most of the books I start reading, I finish within a rather short period of time.


##### Previous reading-related flow experiences

In order to identify readers generally prone to flow experiences, participants were inquired about previous reading-related flow experiences. Therefore, a flow state in fiction reading was described to them by systematically transferring flow components to this context:

There are readers, who have the feeling to fully immerse in the activity during reading. Then, they block out themselves, their everyday life, and their surroundings for a certain period of time and fully concentrate on reading. They become so focused that they lose track of time and forget everything around them. It seems like they melt with the story during reading.

The story for its part becomes accessible for them almost by itself. The readers intuitively comprehend what the story is about. Neither do they need to actively think about the text nor are they thinking about other things while they are reading. Reading and comprehending the text does not seem very exhausting to them, as if they could read on for hours without any problems. They feel that the story is clear, understandable, and entertaining for them and that reading this text runs smoothly and fluently.

All in all, these readers fell neither bored nor stressed, but rather optimally challenged. They know, that they will be able to emphasize and comprehend the story and that the book has sufficient quality to let them have a good time with it. They enjoy reading and are therefore highly motivated to read on in the book and to again and again engage in reading during their leisure time.

Based on this description, participants indicated in how far they had ever experienced such a state during fiction reading, how frequently they would normally experience it, and whether they consider this a typical reading experience on five-point Likert-type response scales. Quality and frequency of as well as proneness to past reading-related flow experiences were expected to show positive associations with flow during reading in the study as measured with the RFSS flow score.

#### Discriminant Measures

In order to contrast flow in fiction reading with other common pleasure-related narrative engagement concepts, presence, identification, suspense, and cognitive mastery were measured using specially developed short scales. Existing measures were not considered appropriate for the purpose of this study due to their long-scale format and multi-dimensional conceptualization, which would have led to a problematic degree of shared item content across scales. To avoid inflating correlations, each narrative engagement concepts was instead measured with items carefully chosen to represent its unique qualities only. For instance, the definition of identification as an imaginative process by which the characters’ perspectives are internalized (Cohen, 2001) does not specifically entail absorption; however, readers who are absorbed in a story seem more likely to identify with its characters and readers that identify themselves with a character seem more likely to get absorbed in a story, so that both states presumably often coincide in fiction reading. In order to ensure accurate measurement and thus understanding of the interaction of different narrative engagement concepts in reading pleasure, it is nevertheless important to use scales that are highly specific to the concept in question. That is, a scale to measure identification in fiction reading should not include absorption-related items, since absorption is by definition not part of this concept, to avoid creating artificial conceptual overlap. Bearing in mind this need for highly concept-specific measurement, we opted for rationally constructed *ad hoc* measures of presence, identification, suspense, and cognitive mastery instead of using existing less-specific scales.

For assessing presence, five items (ω = 0.79 [0.73, 0.83]) were adapted from the Telepresence Scale (Kim and Biocca, 1997) and the Narrative Engagement Scale (Busselle and Bilandzic, 2009). To measure identification, eight items were taken from the Identification Scale by Cohen (2001) (ω = 0.90 [0.86, 0.93]). With regard to suspense, a five-item scale (ω = 0.84 [0.79, 0.88]) was developed building on Knobloch et al. (2004) measurement approach. Cognitive mastery was assessed using a six-items scale (ω = 0.89 [0.85, 0.91]) adapted from items developed to measure narrative comprehension (Kuijpers, 2014) and eudaimonia (Oliver and Raney, 2011). For all items, a seven-point Likert-type scale ranging from *strongly disagree* to *strongly agree* was employed. A full list of the items used can be found in Table 2.

#### Criterion

To assess perceived degrees of individual reader skills and text challenge in relation to the self-selected novel, we adapted two supplemental items from the FSS (Rheinberg et al., 2003): Item A “*I think my skills for reading and comprehending this book are... too low/just right/too high.”*; item B “*For me personally, the degree of challenge that this book poses on the reader is... too low/just right/too high.”* The middle category of the seven-point Likert-type response scales indicated perceived optimal balance. Since optimal balance of skills and challenges is the central pre-condition for flow, the RFSS flow score was expected to be predictable by self-report ratings on these two items.

### Statistical Analysis

All analyses described in the following were performed using the statistical software program R v3.3.1 (R Core Team, 2016).

#### Exploratory Factor Analysis

Due to substantial item rewording and different domain of application, we did not expect the RFSS to fully replicate the original FSS’s factorial structure. Therefore, we chose exploratory factor analysis (EFA) to test the dimensionality of the RFSS. Given the highly skewed distributions of responses, we conducted an EFA for ordered-categorical indicators based on a polychoric correlations coefficients matrix. For conducting the EFA, the principal axes factor analysis method and a maximum likelihood estimator were employed. In order to determine the number of factors to be extracted, we used parallel analysis, Velicer’s MAP test, and a scree test. Subsequently, the indicated number of factors was extracted using an oblique Geomin-type rotation since different subdimensions of flow have been shown to intercorrelate (Rheinberg et al., 2003).

#### Validity Analysis

Based on the assumption derived from flow theory that RFSS flow scores should peak when participants report to perceive an optimal balance of skills and challenges, criterion validity was tested regressing RFSS flow scores on skills-challenge balance using polynomial regression models. To investigate convergent validity, Spearman and point-biseral correlations between RFSS flow scores and theoretically flow-related reading and reader variables were calculated. Discriminant validity was explored by calculating Spearman correlations between RFSS flow scores and presence, identification, suspense, and cognitive mastery scores. In order to confirm that RFSS items load on a distinct latent variable than items assessing presence, identification, suspense, or cognitive mastery, single-factor confirmatory factor analysis (CFA) models for ordered categorical indicators were contrasted with corresponding multi-factor models. For all CFAs a robust weighted mean- and variance-adjusted least squares estimator (WLSMV) was used, which outperforms other estimators in case of skewed data distributions (Flora and Curran, 2004). Model fit was regarded as acceptable if the Tucker-Lewis index (TLI) was above 0.95, the comparative fit index (CFI) above 0.96, and the root mean square error of approximation (RMSEA) close to 0.05 (Yu, 2002).

## Results

### Factorial Structure of the RFSS

The 10 RFSS items were subjected to an EFA using the principal axes factor analysis method and a maximum likelihood estimator based on polychoric correlations coefficients. Indicating sufficiently strong relationships among items, the Kaiser-Meyer-Olkin measure of sampling adequacy was 0.82. While the parallel analysis turned out inconclusive as a potential third factor lay within the error bar of 50 iterations, Velicer’s MAP test suggested the extraction of two factors, which was further supported by employing a scree test. Solutions for both two and three factors were examined using oblique Geomin-type rotation. The two-factor solution was preferred as it is supported by flow theory as well as by the original FSS’s factor structure, whereas the three-factor solution did not yield interpretable results. The Kaiser index of factorial simplicity (Kaiser, 1974) for the two-factor solution was 0.95, indicating a high tendency toward unifactoriality of loadings and thus further supporting this solution.

The two-factor solution, explaining 45% of the variance, showed a simple structure (Table 1) with clear loadings for eight items. Substantial cross-loadings of items 4 and 5 indicated ambiguous item-factor associations, which lead to the removal of these items, reducing the RFSS to eight items in total. The two factors, the remaining items loaded on, overall correspond with the Absorption and Smooth Processing subscales of the FSS, from which the RFSS was derived, and show an estimated correlation of *r* = 0.55, indicating closely related yet distinct facets of flow in reading.

To test whether the calculation of a global flow score across subscales, as indicated by the theoretical assumption of flow being a higher-order concept for a multi-dimensional state, is psychometrically justified, a CFA model with Flow as a higher-order factor needs to be conducted. This model, however, is not identified with two first-order factors only (Absorption and Smooth Processing), rendering it impossible to provide clear empirical evidence in favor or against the global flow score. We therefore tested a first-order CFA model in which all items load on just one factor (Flow), which is the closest possible approximation to testing the presumption of a global factor. The single-factor model did not show acceptable fit to the data (TLI = 0.817, CFI = 0.869, RMSEA = 0.212), further supporting the use of a two-factor model as indicated by the EFA. In the following, we report results for both the empirically validated subscale scores and the theoretically indicated global flow score.

### Scale Scores and Reliability

Individual RFSS flow scores were calculated by averaging item scores by participants. The mean RFSS global flow and subscale scores (see Table 3) in the sample were all above the response scale’s midpoint, indicating that, on average, participants did experience flow during 20 min of fiction reading. Kruskal-Wallis tests for each score revealed that neither sex nor educational background had a significant effect on flow experiences (for the corresponding subsample’s scores, see Table 3).

**Table 3:** Mean scores and SD for flow, presence, identification, suspense, cognitive mastery, and reading self-efficacy.

Concept	Total sample		Females		Males		Academics		Non-academics	
					
	*Mdn*	*M (SD)*	*Mdn*	*M (SD)*	*Mdn*	*M (SD)*	*Mdn*	*M (SD)*	*Mdn*	*M (SD)*
Global flow	5.6	5.5 (0.9)	5.6	5.6 (0.9)	5.4	5.4 (0.8)	5.5	5.4 (0.09)	5.8	5.6 (0.8)
Absorption	5.2	5.2 (1.1)	5.4	5.3 (1.1)	5.2	5.1 (0.9)	5.1	5.2 (1.1)	5.6	5.4 (1.1)
Smooth Processing	6.3	6.0 (1.0)	6.3	6.1 (1.1)	6.0	5.8 (1.0)	6.3	5.9 (1.1)	6.3	6.1 (0.9)
Presence	5.3	5.2 (1.0)	5.3	5.1 (1.0)	5.2	5.0 (1.0)	5.2	5.1 (1.1)	5.3	5.3 (0.9)
Identification	5.3	5.2 (1.0)	5.4	5.3 (1.0)	4.9	4.9 (1.1)	5.1	5.1 (1.0)	5.5	5.4 (1.0)
Suspense	5.4	5.3 (1.2)	5.6	5.4 (1.2)	5.0	4.9 (1.2)	5.2	5.0 (1.2)	5.8	5.6 (1.1)
Cognitive mastery	4.5	4.4 (1.4)	4.5	4.4 (1.4)	4.5	4.3 (1.4)	4.6	4.4 (1.4)	4.3	4.4 (1.4)
Reading self-efficacy	4.3	4.2 (0.6)	4.3	4.2 (0.6)	4.3	4.1 (0.6)	4.3	4.2 (0.6)	4.3	4.2 (0.6)


**N* = 229; *n* = 181 for females and *n* = 45 for males (*n* = 3 no response); *n* = 134 for academics and *n* = 92 for non-academics (*n* = 3 no response); significant effect of gender [χ^*2*^(2) = 6.25, *p* < 0.05] and educational background [H (2) = 15.74, *p* < 0.001] only for suspense; flow, presence, identification, suspense, and cognitive mastery were measured on a 7-point, reading self-efficacy on a 5-point Likert-type scale.*

Composite reliability was calculated for both the RFSS’ subscale scores and for the global flow score, yielding reliability estimates of ω = 0.77 [0.68, 0.82] for Smooth Processing, ω = 0.80 [0.75, 0.85] for Absorption, and ω = 0.89 [0.85, 0.95] for the RFSS flow score. Thus, the scale’s reliability estimates significantly exceed the commonly reported cut-off value of 0.70 (Nunnally and Bernstein, 1994; Ponterotto and Charter, 2009), which is usually accepted as sufficient for group testing.

### Validity

#### Construct Validity

##### Convergent validity

Table 4 shows Spearman correlations of the RFSS flow score and both its Absorption and Smooth Processing subscale scores with reading and reader variables as well as with variables related to previous reading-related flow experiences. As expected based on flow theory and results from general flow research, the RFSS scores showed positive associations to these variables.

**Table 4:** Correlations of RFSS scale scores with reading and reader variables, previous reading-related flow experiences, and concepts of pleasure-related narrative engagement.

	RFSS scale score		
	
Measure	Absorption	Smooth Processing	Global flow
Reading and reader variables			
Reading pleasure	0.49	0.26	0.48
Motivation to read on in the novel	0.52	0.32	0.53
Motivation to read similar novel	0.23	0.13	0.23
Reading affinity	0.12	0.12	0.14
Frequency of reading fictional texts	0.13	0.21	0.18
Reading-related self-efficacy	0.23	0.20	0.25
Previous reading-related flow experiences			
Quality of past flow in fiction reading	0.27	0.20	0.28
Frequency of flow in fictional texts	0.27	0.15	0.33
Proneness to flow in fiction reading	0.34	0.25	0.35
Concepts of pleasure-related narrative engagement			
Presence	0.74	0.30	0.69
Identification	0.65	0.44	0.68
Suspense	0.70	0.41	0.71
Cognitive mastery	0.28	-0.10	0.19


**N* = 229; correlations above 0.13 are statistically significant with *p* < 0.05.*

##### Discriminant validity

As can be seen in Table 4, Spearman correlations of RFSS flow scores with presence, identification, and suspense scores revealed strong associations between these concepts, and a medium-level association between flow and cognitive mastery. Given that most of the correlations indicate approximately 50% of shared variance, we turned to CFA modeling to test whether flow as measured with the RFSS was still empirically distinguishable from presence, identification, suspense, and cognitive mastery. If that was the case, a multi-factor CFA model with independent clusters and freely correlating latent variables, which includes both items from the RFSS and items assessing one of these other concepts, should show better data-fit than the corresponding unidimensional CFA model; thus, the former CFA model indicates two different latent variables underlying the two measures, whereas the latter indicates a single latent variable.

**FIGURE 1:**
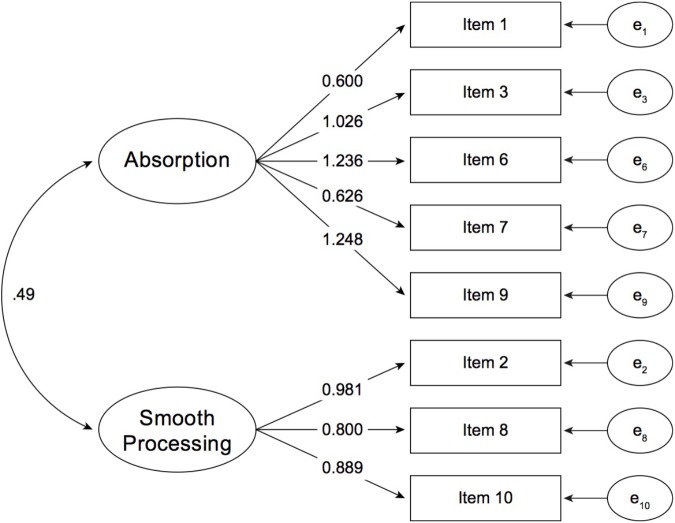
Results of the confirmatory factor analysis for the Reading Flow Short Scale (RFSS). Two latent variables, Absorption and Smooth Processing, explain the variability in item responses; ^∗∗∗^*p* < 0.001.

This assumption was tested for the RFSS paired with presence, identification, suspense, and cognitive mastery, respectively. In a first step, separate CFA models for each concept (for flow see Figure 1) were conducted, based on which, in a second step, CFAs for all pairings followed.

As can be seen in Table 5, the multi-factor model representing independent clusters for different measures showed better data-fit than the corresponding single-factor model for each construct pairing. Satorra-Bentler corrected scaled *χ*^2^ difference tests confirmed that the multi-factor models indicating separable latent variables significantly outperformed the single-factor models (see Table 5).

**Table 5:** Confirmatory factor analyses results and model comparisons for flow and other pleasure-related reading engagement concepts.

Construct	Model	Fit indices					Model comparisons		
			
		*χ*^2^ (*df*)	*p*	TLI	CFI	RMSEA	Δ *χ*^2^	*df*	*p*
Flow	Two-factor^a^	20.49 (18)	0.365	0.993	0.995	0.008			
Presence	Single-factor	3.04 (3)	0.385	1.000	1.000	0.057			
Identification	Two-factor^b^	26.73 (18)	0.084	0.992	0.995	0.046			
Suspense	Single-factor	4.04 (3)	0.258	0.997	0.999	0.039			
Cognitive mastery	Single-factor	6.68 (8)	0.572	1.001	1.000	0.000			
Flow and presence									
	Three-factor	124.64 (60)	<0.001	0.971	0.977	0.069			
	Single-factor	361.95 (62)	<0.001	0.868	0.895	0.146			
							75.50	2	<0.001
Flow and identification									
	Four-factor	191.13 (97)	<0.001	0.964	0.971	0.065			
	Single-factor	516.76 (103)	<0.001	0.849	0.871	0.133			
							106.03	6	<0.001
Flow and suspense									
	Three-factor	149.83 (60)	<0.001	0.965	0.973	0.081			
	Single-factor	335.51 (62)	<0.001	0.897	0.918	0.139			
							60.96	2	<0.001
Flow and cognitive mastery									
	Three-factor	161.06 (73)	<0.001	0.965	0.972	0.073			
	Single-factor	1262.06 (76)	<0.001	0.548	0.623	0.262			
							157.04	3	<0.001


*^*a*^Comprising absorption and smooth processing; ^*b*^comprising cognitive perspective taking and empathy; TLI, Tucker-Lewis index; CFI, comparative fit index; RMSEA, root-mean-square error of approximation; Δ χ^*2*^, test statistic of the Satorra-Bentler corrected scaled χ^*2*^ difference test; *df*, degrees of freedom.*

#### Criterion Validity

Following the rationale of flow theory, we regressed the RFSS global flow and subscale scores on measures of perceived skills-challenge balance. Since flow theoretically results from optimally balanced skills and challenges, we expected an inverted U-shaped association between the RFSS scores and responses on the items assessing skills and challenge, for which the response scales’ midpoints represent optimal balance. We combined RFSS scores into four categories to obtain sufficient data points per category and then conducted a second-order polynomial regression model for ordered categorical outcome variables.

**FIGURE 2:**
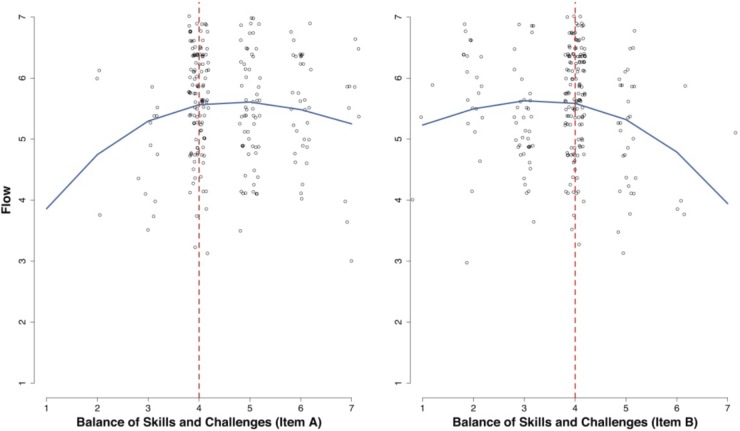
Regression of the RFSS flow scores on balance of skills and challenges. For item A, observations right and, for item B, left of the vertical dashed line indicate perception of low challenge. Individual observations are shown as jittered hollow circles.

As expected for the global flow score, significant positive linear (coefficient *b*_1_) and negative quadratic (coefficient *b*_2_) effects were found for both items measuring skill-challenge balance (item A: *b_1_* = 1.89, *z* = 1.98, *p* = 0.048*, b_2_* = -0.19, *z* = -1.98, *p* = 0.048; item B: *b_1_* = 1.32, *z* = 1.98, *p* = 0.048*, b_2_* = -0.21, *z* = -2.21, *p* = 0.027). Observations and regression lines are illustrated in Figure 2.

For the Absorption subscale score, both linear and quadratic effects were non-significant, while for Smooth Processing, only the quadratic effect for item B gained significance (*b_2_* = -0.29, *z* = -2.87, *p* = 0.004).

## Discussion

Against the background of psychometrically limited methods for flow measurement in fiction reading, we developed the RFSS, an 8-item reading-specific flow scale based on a well-established general flow scale, the FSS (Rheinberg et al., 2003), and its componential measurement approach. Our study provides evidence that the RFSS is a useful instrument for assessing flow states in fiction reading. Thus, the scale shows (1) a conceptually adequate factorial structure and good reliability estimates, (2) the predicted relationship with perceived skills-challenge-balance, (3) associations with theoretically flow-related concepts, and (4), on top of substantial convergence, also sufficient distinctness when compared to other concepts of pleasure-related narrative engagement.

In support of a successful adaptation of the FSS, RFSS items loaded on two factors, largely corresponding to the two FSS subscales Absorption and Smooth Processing. However, two items which showed no clear loading pattern had to be discarded. Since item 4 (“I had no problem to concentrate during reading.”) comprises both the notion of subjective effortlessness indicating Smooth Processing and of high concentration indicating Absorption, and since item 5 (“My mind was totally clear during reading.”) is phrased in a way that allows for various interpretations, cross-loadings are explicable and the decision to remove those items seems justified. Another three items did not load on the same factor as their FSS counterparts. Given that the FSS and RFSS differ both in the domain of application and in item wording, such minor alterations in the factorial structure were to be expected. As a result, however, the RFSS Absorption dimension ended up being over-represented compared to the Absorption dimension on the original FSS. Following Rheinberg and Vollmeyer’s (2001) assumption that facets of flow differ in weight across activities, one could speculate that flow in fiction reading is indeed primarily characterized by absorption, while the role of smooth processing is smaller in this context compared to other flow activities.

In any case, smooth processing and absorption flow components are theorized to add up to the specific state of flow (Rheinberg et al., 2003), so that the calculation of a global flow score seems indicated. However, unlike larger multi-dimensional flow scales (Jackson and Marsh, 1996; Jackson et al., 2008), the original FSS as well as the RFSS show a two-factor structure, which does not allow psychometric testing of a higher-order model involving a second-order factor representative of global flow. Thus, the higher order model is not identified with two first-order factors only, rendering it impossible to provide evidence in favor or against calculating a global flow score. The insufficient data-fit of a single-factor solution supports the multidimensional conceptualization of flow and the calculation of subscale scores, but cannot provide clarification regarding the global flow score. In the absence of clear empirical evidence, the decision to report a global flow score when using the RFSS can only be based on considerations of feasibility and practical application, its widespread use in flow literature, also for the original FSS (Rheinberg et al., 2003), and on empirical indicators such as the correlation between subscales and internal consistency.

To further validate the RFSS, the relationship between flow as measured by this scale and the flow-criterion of perceived optimally balanced challenges was examined. In line with flow theory, readers who perceived their respective text’s level of challenge as optimally fitting to their skills scored high on the RFSS in terms of the global flow score, but not in terms of the subscale scores. These results indicate that while absorption and smooth processing independently of one another show different associations to perceived text challenge, the combination of both high absorption and smooth processing, which characterizes a flow state, can only be found for texts that pose a certain, optimal degree of challenge.

A closer examination of this association between skills-challenge balance and the global flow score, however, revealed that flow was also high for texts perceived as slightly less than optimally challenging. This finding could be a methodological artifact, since the corresponding self-reports might suffer from the difficulty to intuitively estimate skills-challenge balance in fiction reading and from potential biases toward a more flattering intellectual self-presentation. On the other hand, flow might indeed not be limited to reading books perceived as optimally challenging only: A meta-analysis of 28 studies found flow to occur mostly, but by no means exclusively under optimally challenging conditions (Fong et al., 2014). In line with this result, the association between flow and optimal challenge levels has been shown to underlie situational and individual influences (Keller and Landhäußer, 2012). Following this rationale, books that pose a less than optimal challenge level on the reader can still be appealing to certain types of readers or become appealing under certain circumstances: For instance, a reader normally interested in challenging material, might pick up and enjoy a young-adult book when reading for relaxation purposes. Whether fiction reading is an activity specifically associated with situations or individuals that facilitate flow experiences under less than optimally challenging conditions, remains an interesting open question for future research.

Based on flow theory and research in other activities, we expected flow in fiction reading to be positively associated with intrinsic reading enjoyment (i.e., Keller and Bless, 2008; Keller and Blomann, 2008), heightened reading-related self-efficacy (Rheinberg et al., 2003), and general affinity toward reading (Csikszentmihalyi, 1975) as well as with a tendency to repeatedly engage in reading (Csikszentmihalyi, 1975). The corresponding correlations obtained in our study were all in the expected direction, as were the correlations between RFSS flow scores and measures of previous reading-related flow experiences. However, correlations were moderate in size. This might result from limited measurement reliability as most concepts were assessed with single-items. Correlations with measures of previous reading-related flow experiences could be particularly affected by methodological limitations, as these measures were based on a description of flow in fiction reading that had not been pre-tested or psychometrically analyzed itself. For most constructs, correlations were higher with Absorption than with Smooth Processing scores. Therefore, it is also possible that global flow score correlations were artificially diminished as a consequence of the smooth processing part of flow being under-represented in the RFSS.

Conversely, correlations between RFSS flow scores and presence, identification, suspense, and cognitive mastery scores were overall high. This was to be expected, as all of the concepts share a strong relationship with reading pleasure. Importantly, CFA modeling empirically confirmed that there is still sufficient distinctness between these concepts within the global reading experience. Thus, flow in fiction reading goes beyond other concepts of pleasure-related narrative engagement, opening a new perspective for reading pleasure research. The high average flow score found in this study supports both the idea of flow being a regular reading state, and of fiction reading promoting flow. Given the close link between flow and intrinsic enjoyment and the comprehensive framework of flow theory, this concept is of considerable added value for research on reading pleasure.

To overcome the limitations of the current study, future research should replicate the psychometric properties of the RFSS with more representative samples of readers and novels. Given that the main aim of the study was to develop a reading-specific flow measure, we deliberately chose to recruit people prone to flow experiences in reading by advertising the online survey in bookstores and amongst literature students. However, this specific sample showed a high educational background and gender bias, so that the current results’ generalizability needs to be tested with additional samples. Moreover, future studies should include controlled laboratory conditions and objective measurement approaches. Since retrospective self-report state measures like the RFSS are not free from bias, assessments from other domains, such as psychophysiological or eye-tracking measures, could prove an important complement.

Nevertheless, the RFSS significantly adds to the portfolio of measurement instruments in reading research, as it is the first theoretically derived and psychometrically evaluated measure of flow during fiction reading, opening new perspectives to explore reading pleasure evolvement, be it in leisure, school, or therapeutic contexts. Moreover, the current study’s results encourage further investigations of the nature of flow experience in different activity contexts and the possibilities to measure flow with activity-specific scales. By comparing the characteristic pattern of flow components and their interaction in creating an overall flow state across different activities, more insight can be gained about the flow concept in general. Looking into flow states in mental activities emphasizing absorption, such as reading, complements the wide branch of flow research that focuses on motoric or competitive activities emphasizing smooth processing, which will eventually allow for a more complete picture of flow.

## Ethics Statement

This study was carried out in accordance with the recommendations of guidelines and rules of the Max Planck Society. The procedure was approved by the Ethics Council of the Max Planck Society. All subjects gave informed consent in accordance with the Declaration of Helsinki.

## Author Contributions

BT and WS designed the study. BT collected data, wrote the first draft of the manuscript, and developed and performed the statistical analysis in conjunction with WS. WS and WM reviewed and edited the manuscript and approved the final version of the manuscript.

## Conflict of Interest Statement

The authors declare that the research was conducted in the absence of any commercial or financial relationships that could be construed as a potential conflict of interest.
